# Soluble TREM2 levels associate with conversion from mild cognitive impairment to Alzheimer’s disease

**DOI:** 10.1172/JCI158708

**Published:** 2022-12-15

**Authors:** Aonan Zhao, Yang Jiao, Guanyu Ye, Wenyan Kang, Lan Tan, Yuanyuan Li, Yulei Deng, Jun Liu, for the Alzheimer’s Disease Neuroimaging Initiative (ADNI)

**Affiliations:** 1 Department of Neurology and Institute of Neurology, Ruijin Hospital affiliated with the Shanghai Jiaotong University School of Medicine, Shanghai, China.; 2 Department of Neurology, Qingdao Municipal Hospital, Qingdao University, Qingdao, Shandong Province, China.; 3 Department of Neurology, Ruijin Hospital/LuWan Branch, affiliated with the Shanghai Jiaotong University School of Medicine, Shanghai, China.; 4 CAS Center for Excellence in Brain Science and Intelligence Technology, Ruijin Hospital affiliated with the Shanghai Jiaotong University School of Medicine, Shanghai, China.; 5 The ADNI is detailed in Supplemental Acknowledgments.

**Keywords:** Inflammation, Neuroscience, Alzheimer disease, Diagnostics, Neurodegeneration

## Abstract

**BACKGROUND:**

Soluble triggering receptor expressed on myeloid cells 2 (sTREM2) plays an important role in the clearance of pathological amyloid-β (Aβ) in Alzheimer’s disease (AD). This study aimed to explore sTREM2 as a central and peripheral predictor of the conversion from mild cognitive impairment (MCI) to AD.

**METHODS:**

sTREM2 and Aβ_1–42_ levels in cerebrospinal fluid (CSF) and florbetapir-PET (AV45) images were analyzed for healthy control (HCs), patients with MCI, and patients with AD from the ADNI database. Peripheral plasma sTREM2 and Aβ_1–42_ levels were determined for our Neurology database of Ruijin Hospital for Alzheimer’s Disease (NRHAD) cohort, and patients with MCI were reevaluated at follow-up visits to assess for progression to AD. The association between CSF and plasma sTREM2 levels was analyzed in data from the Chinese Alzheimer’s Biomarker and Lifestyle (CABLE) database.

**RESULTS:**

The results showed that patients with MCI who had low levels of CSF sTREM2 and Aβ_1–42_ were more likely to develop AD. Among participants with positive Aβ deposition, as assessed by AV45 imaging, elevated CSF sTREM2 levels were associated with a decreased risk of MCI-to-AD conversion. Meanwhile, in the NRHAD cohort, individuals in the MCI group with high sTREM2 levels in plasma were at a greater risk for AD, whereas low Aβ_1–42_ with high sTREM2 levels in plasma were associated with a faster cognitive decline. In addition, CSF sTREM2 levels were highly correlated with plasma sTREM2 levels in the CABLE database.

**CONCLUSION:**

These findings suggest that sTREM2 may be useful as a potential predictive biomarker of MCI-to-AD conversion.

**FUNDING:**

This study was supported by grants from the National Natural Science Foundation of China (grant nos. 82001341, 82071415, 81873778, and 82201392); the Shanghai Sailing Program (grant no. 22YF1425100); and the China Postdoctoral Science Foundation funded project (grant no. 2021M702169).

## Introduction

Triggering receptor expressed on myeloid cells 2 (TREM2), a transmembrane receptor expressed in myeloid cells, plays an important role in regulating microglial phagocytosis and the response to inflammatory stimuli (1–3). The extracellular domain of TREM2 can be cleaved to soluble TREM2 (sTREM2) and released into the cerebrospinal fluid (CSF) (4–8) and is regarded as an indicator of microglial activity (9, 10). Microglial cells are essential for the recognition and clearance of pathological amyloid-β (Aβ) in Alzheimer’s disease (AD), and sTREM2 mediates various microglial responses to Aβ during neurodegeneration and regulates microglia-plaque interaction (11–13).

Studies in AD animal models confirmed that sTREM2 exerts a protective effect in Aβ pathology in AD models (14, 15). However, discordant results have been reported on the relationship between sTREM2 and Aβ in clinical studies. One study showed that, in the CSF of patients with mild cognitive impairment (MCI), sTREM2 levels were correlated with Aβ_1–42_ levels (16). In contrast, other studies reported that, in AD, CSF sTREM2 levels were correlated with CSF Tau rather than Aβ_1–42_ levels (14, 16, 17). Thus, in-depth research is needed to clarify the clinical significance of sTREM2 as a potential central and peripheral biomarker for AD.

Furthermore, a recent study showed that sTREM2 and the ratio of sTREM2 to phosphorylated Tau (sTREM2/p-Tau) in the CSF is associated with cognitive decline (18). Previous studies have shown that the concentration of sTREM2 changes dynamically during AD progression and peaks at the early symptomatic stage (19, 20), suggesting that the early increase in CSF sTREM2 could be used to predict the MCI-to-AD conversion. Although several studies have shown that higher levels of *TREM2* mRNA in PBMCs are associated with MCI-to-AD conversion (19, 21–24), the predictive power of plasma sTREM2 from the peripheral blood is understudied.

**Figure 1:**
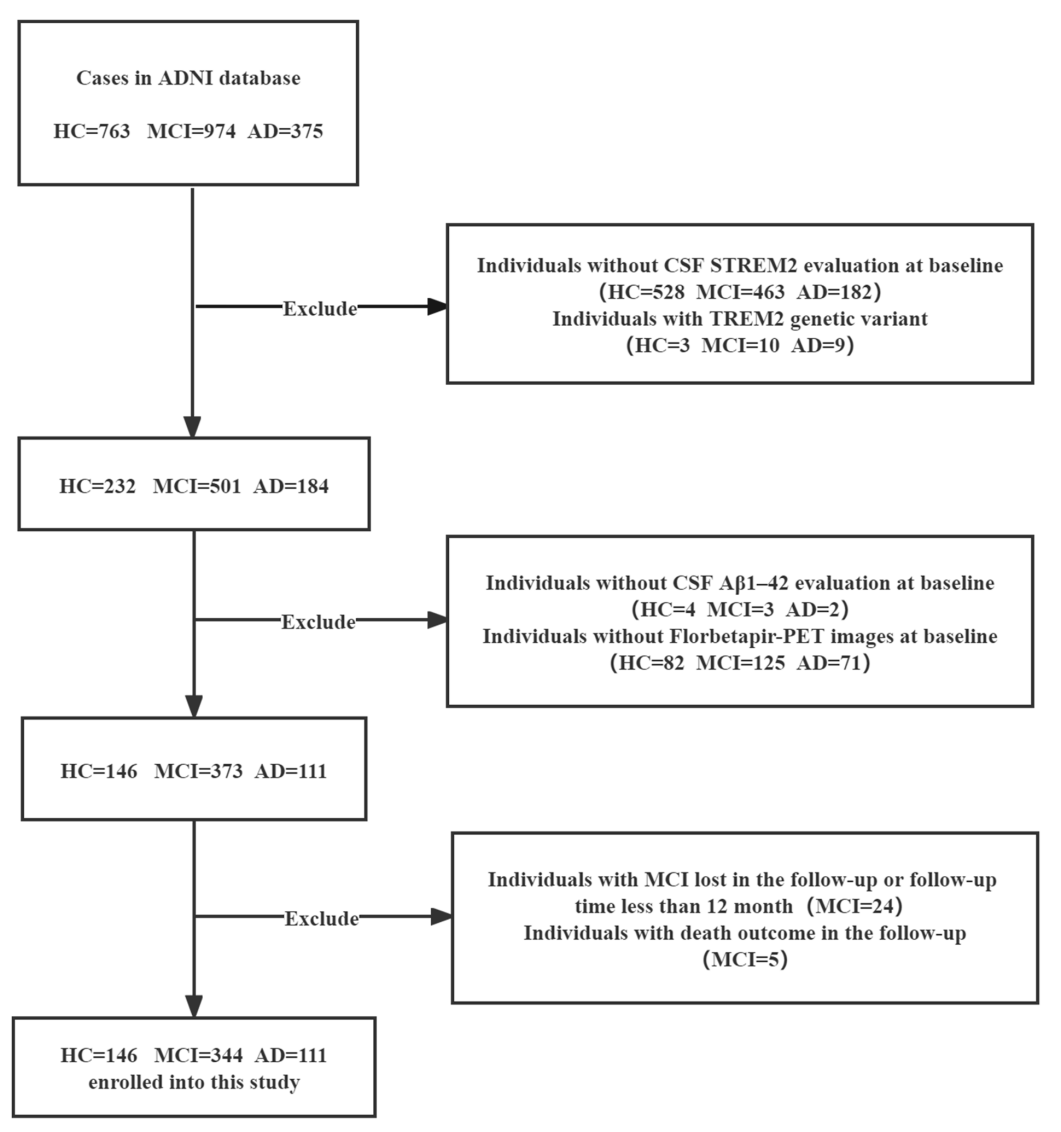
Flow chart of the study population selection from ADNI database including exclusion criteria.

Hence, we first investigated the altered CSF sTREM2 concentration in different subgroups of individuals classified by CSF Aβ_1–42_ levels and ^18^F-labeled radioligand PET imaging (AV45) findings from the ADNI database in a cross-sectional, longitudinal cohort study (Figure 1). Moreover, to explore the potential role of peripheral blood sTREM2 in MCI-to-AD conversion, we studied whether plasma sTREM2 could predict the cognitive decline in patients with MCI and assessed the presence of amyloid pathology in our clinical cohort.

## Results

### Baseline demographic and clinical characteristics of participants from the ADNI database.

Demographic and clinical features of the healthy controls (HCs), patients with MCI, and patients with AD are listed in Table 1. No significant differences were observed among the study participants with regard to age or sex. However, we noted a distinct difference between the 3 groups in terms of their years of education, with patients with AD having fewer years of education than HCs (*P* < 0.001). Differences in cognitive function, assessed by the Mini–Mental State Examination (MMSE) and the Montreal Cognitive Assessment (MoCA), were detected between the groups, with both AD (*P* < 0.001) and MCI (*P* < 0.001) patients having lower scores than HCs. Furthermore, patients with MCI or AD showed functional activity impairment, as assessed by a functional activities questionnaire (FAQ), compared with HCs (both *P* < 0.001, Table 1). The CSF levels of sTREM2 and Aβ_1–42_ are also shown in Table 1. There was no significant difference in sTREM2 levels between the 3 groups, while the concentration of Aβ_1–42_ was significantly decreased in patients with MCI and patients with AD compared with HCs (1,022.8 ± 379.9 pg/mL for HC; 888.6 ± 339.1 pg/mL for MCI; 640.8 ± 252.9 pg/mL for AD, *P* < 0.001). MCI and AD patients showed higher CSF total Tau (t-Tau) levels and p-Tau levels than did HCs (*P* < 0.001). Moreover, the standardized uptake value ratio (SUVR) of AV45 was higher in MCI and AD patients than in HCs (1.1 ± 0.2 for HCs; 1.2 ± 0.2 for MCI; 1.4 ± 0.2 for AD, *P* < 0.001). On the basis of a cutoff value of 1.11 for distinguishing amyloid positivity (25), we further divided the HC, MCI, and AD groups into 2 subgroups: positive (Aβ^+^/AV45^+^) and negative (Aβ^–^/AV45^–^). Next, the enrolled participants were classified as ApoE ε4 carriers or ApoE ε4 noncarriers, and the results showed that there were more ApoEε4 carriers among AD and MCI patients than among HCs (*P* < 0.001, Table 1).

### ADNI database longitudinal evaluation of MCI converters and nonconverters and risk factors.

Since CSF sTREM2 levels were associated with Aβ pathology and therefore could be predictive of disease progression (26), we evaluated CSF sTREM2 in a longitudinal study. Of 344 patients who were followed up until 2019, a total of 103 (29.9 %) developed definite AD after a mean of 72.8 months (Supplemental Table 1; supplemental material available online with this article; https://doi.org/10.1172/JCI158708DS1). The participants’ sex had an impact on MCI-to-AD conversion, as a significantly larger number of female patients with MCI progressed to AD (*P* = 0.027). We found no significant differences in mean follow-up time, age, ApoE ε4–carrier genotype, or years of education between the groups.

**Figure 2:**
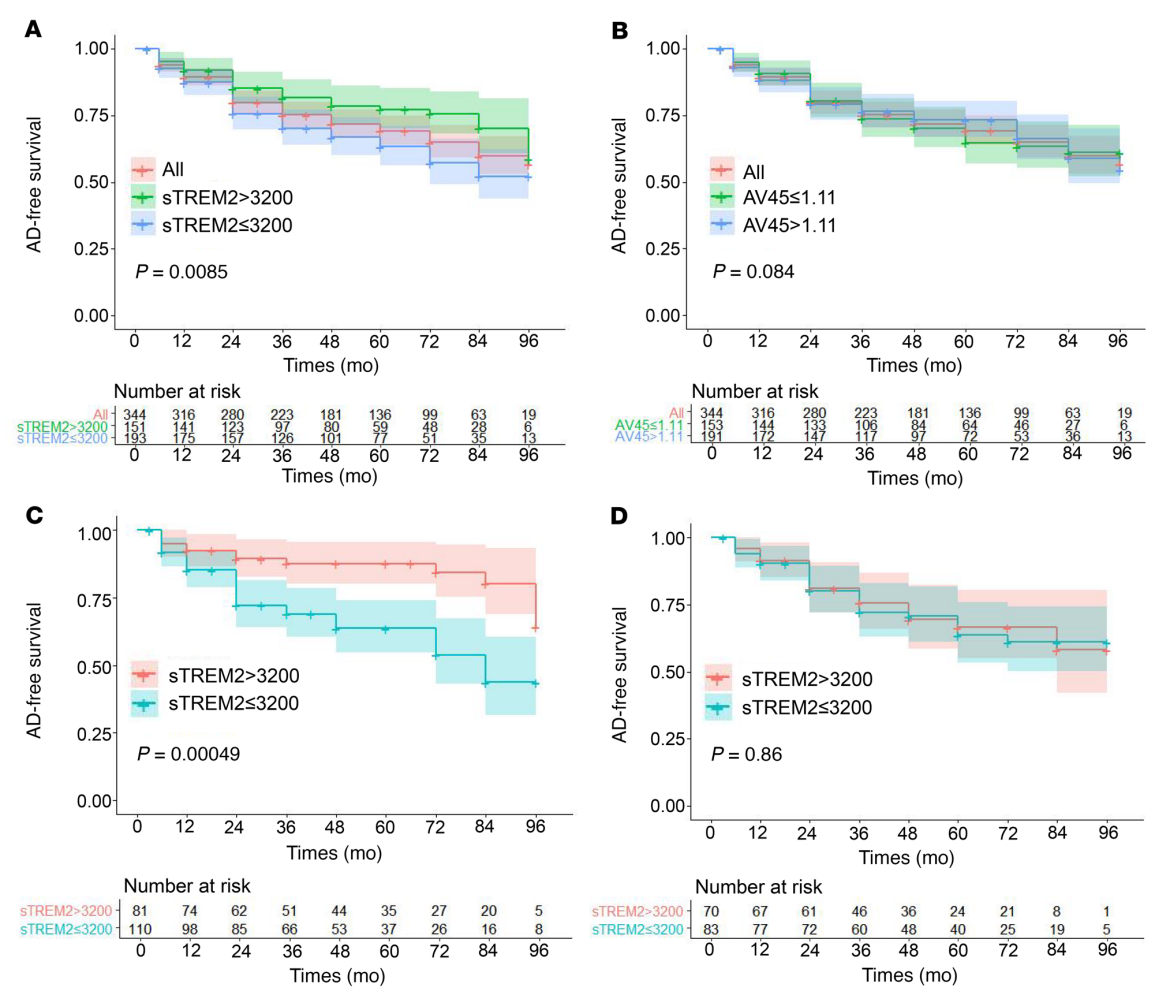
Kaplan-Meier plot of AD-free survival for ADNI database patients with MCI with different CSF sTREM2 levels. (**A**) CSF sTREM2 levels predicted AD-free survival of patients with MCI. (**B**) CSF AV45 values predicted AD-free survival of patients with MCI. (**C**) CSF sTREM2 levels predicted AD-free survival of MCI patients with an AV45 SUVR of 1.11 or higher. (**D**) No significant differences in CSF sTREM2 levels in mean AD-free survival time were observed in patients with MCI who had an AV45 SUVR below 1.11. A total of 146 HCs, 344 patients with MCI, and 111 patients with AD were included for analysis. Data were analyzed using the Kaplan-Meier method.

Furthermore, we applied Cox analysis to evaluate whether CSF sTREM2 levels could predict conversion after adjusting for age, sex, and the p-Tau/t-Tau ratio. As a result, patients with a CSF sTREM2 concentration of less than 3,200 pg/mL (HR = 1.72, *P* = 0.011, Supplemental Table 1) and an Aβ_1–42_ concentration of less than 820.0 pg/mL (HR = 4.35, *P* < 0.001, Supplemental Table 1) were more likely to develop AD. The Kaplan-Meier curves for both sTREM2 levels and AV45 values for all patients with MCI are presented in Figure 2, A and B, respectively. Among patients with MCI who had an MCI AV45 SUVR above 1.11, those with CSF sTREM2 levels of 3,200 pg/mL or lower had a mean AD-free survival of 65.6 months, whereas the mean AD-free survival was 84.8 months for the others (*P* < 0.001; Figure 2C). However, no significant differences in mean AD-free survival were observed in patients with MCI who had an AV45 SUVR below 1.11 (*P* = 0.86; Figure 2D). In addition, Kaplan-Meier analysis indicated a significant difference in mean progression-free survival for patients with CSF Aβ_1–42_ levels below 820.0 pg/mL (*P* = 0.002; Supplemental Figure 1B) and in the remaining population (*P* = 0.011; Supplemental Figure 1C). Next, we investigated potential predictors of disease progression in specific subgroups.

### Evaluation of plasma sTREM2 as a potential biomarker in the NRHAD cohort at baseline.

Demographic features and clinical data for HCs and patients with AD or MCI from our Neurology database of Ruijin Hospital for Alzheimer’s Disease (NRHAD) database are shown in Table 2. There were no significant differences between the 3 groups with regard to age or sex. Furthermore, we classified the enrolled participants as ApoEε4 carriers or ApoE ε4 noncarriers. A larger number of patients with AD or MCI were ApoEε4 carriers relative to HCs (*P* < 0.001, Table 2). Compared with patients with MCI and HCs, patients with AD had lower MMSE, MoCA, and auditory verbal learning test–delayed (AVLT-delayed) scores (*P* < 0.001, Table 2), but had higher Alzheimer’s Disease Assessment Scale–cognitive (ADAS-cog) scores (*P* < 0.001). Patients with AD had higher plasma p-Tau levels than did patients with MCI or HCs (*P* < 0.001, Table 2).

### Validation of plasma sTREM2 in predicting MCI conversion in the NRHAD database.

In total, 90 patients with MCI completed the 3-year follow-up questionnaires, and 23 of 90 patients with MCI (25.6%) developed AD after a median of 3 years of prospective follow-up. Demographic characteristics from the 3-year follow-up visit for MCI converters (MCI-c) and MCI nonconverters (MCI-nc) are described in Supplemental Table 2. There were no significant differences in age, sex, or years of education between the nonconverters and converters.

**Figure 3:**
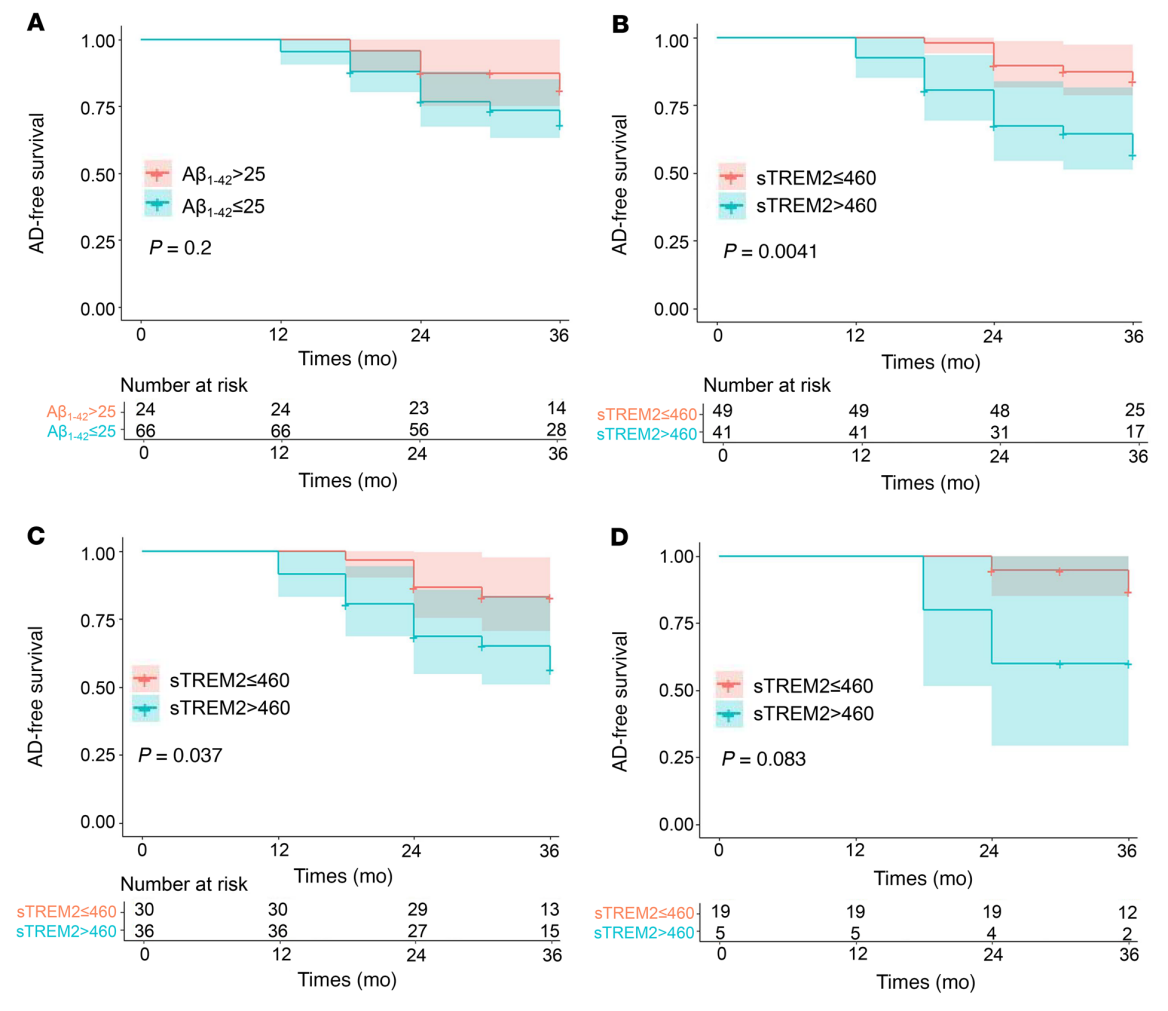
Kaplan-Meier plot of AD-free survival of patients with MCI at the follow-up visit from our NRHAD database. (**A**) No relevant differences were detected in the mean AD-free survival time of patients with MCI with different plasma Aβ_1–42_ levels. (**B**) Plasma sTREM2 levels predicted AD-free survival of patients with MCI. (**C**) Plasma sTREM2 levels predicted AD-free survival of MCI patients with plasma Aβ_1–42_ concentrations of 25 pg/mL or less. (**D**) No difference in plasma sTREM2 levels were detected in the remaining population with plasma Aβ_1–42_ concentrations higher than 25 pg/mL. A total of 104 HCs, 96 patients with MCI, and 96 patients with AD were included for analysis. Data were analyzed by using the Kaplan-Meier method.

Cox proportional hazards regression analysis identified low plasma sTREM2 levels as a risk factor after adjusting for other confounders. The Cox model indicated that patients with plasma sTREM2 levels above 460 pg/mL (HR = 3.45, *P* = 0.008, Supplemental Table 2) and plasma Aβ_1–42_ levels below 25 pg/mL (HR = 8.34, *P* = 0.039, Supplemental Table 2) had a greater risk of incident AD. Kaplan-Meier survival curves revealed no relevant differences in the mean AD-free survival of participants with different Aβ_1–42_ levels (Figure 3A). However, plasma sTREM2 levels were correlated with a significant difference in mean progression-free survival in patients with MCI (*P* = 0.004; Figure 3B), especially for the population with plasma Aβ_1–42_ concentrations below 25 pg/mL (*P* = 0.037; Figure 3C). No difference was detected in the remaining patient population with plasma Aβ_1–42_ concentrations above 25 pg/mL (*P* = 0.083; Figure 3D).

**Figure 4:**
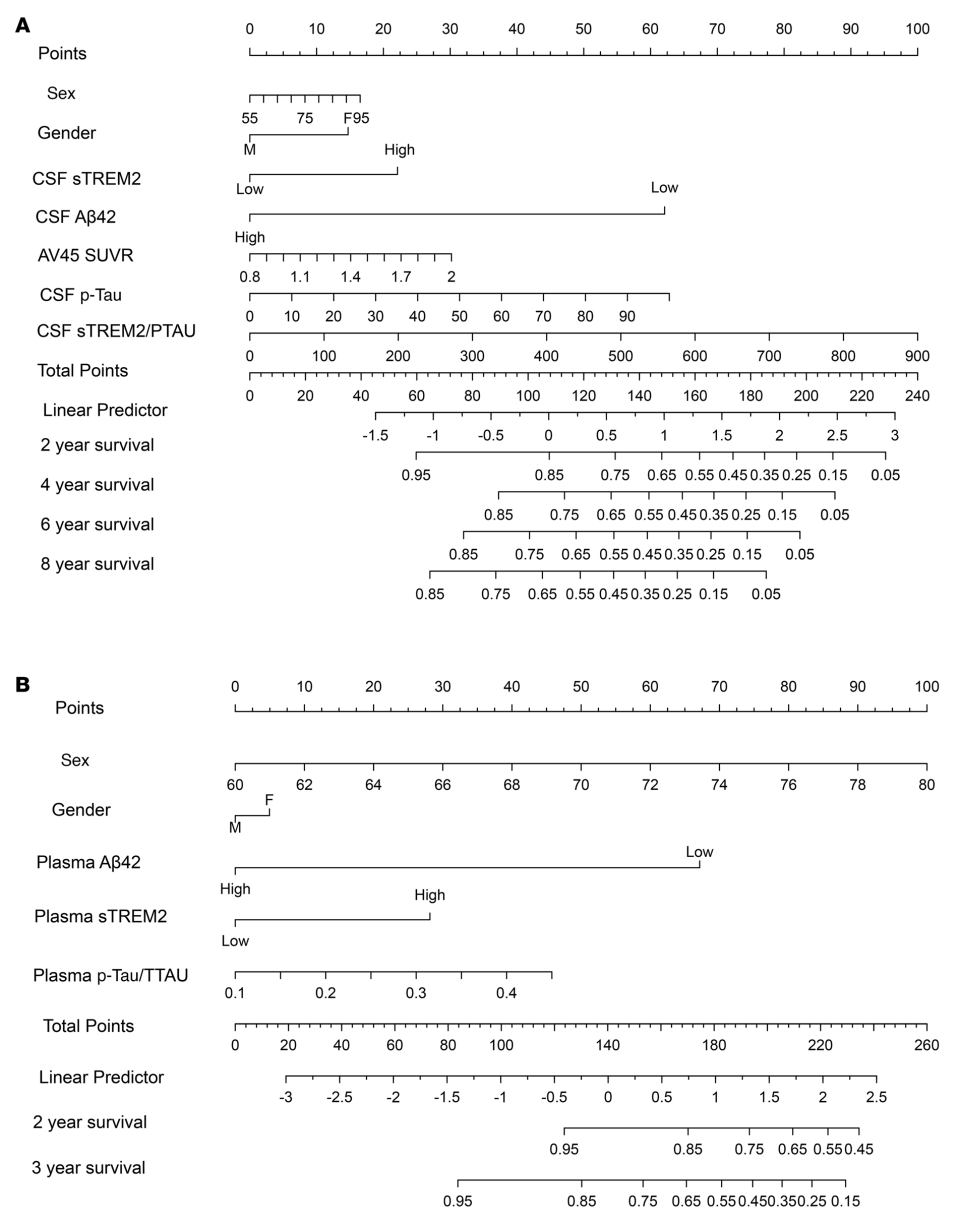
Clinical features nomogram developed for the ADNI and NRHAD databases. (**A**) Predictive nomogram developed from the ADNI cohort, with a C index of 0.968 (95% CI, 0.953–0.982). A total of 146 HCs, 344 patients with MCI, and 111 patients with AD were included for analysis. (**B**) Predictive nomogram from our NRHAD cohort with a C index of 0.935 (95% CI, 0.910–0.967). The linear predictor corresponding to the cumulative points indicated the different possibilities for AD-free survival (shown under the years of follow-up). A total of 104 HCs, 96 patients with MCI, and 96 patients with AD were included for analysis. Multivariable analyses with the Cox proportional hazards model and LASSO analysis were used.

Clinicopathological variables from the ADNI database and our NRHAD clinic cohort were analyzed to formulate a prognostic nomogram of association with MCI conversion. We included the relevant features selected by least absolute shrinkage and selection operator (LASSO) regression in the Cox regression modeling. As shown in Figure 4, sTREM2 levels in both CSF and plasma were identified as independent factors for AD-free survival in patients with MCI. Figure 4A shows the predictive nomogram developed for the ADNI cohort, which had a C index of 0.968 (95% CI, 0.953–0.982). Figure 4B shows the construction of the predictive nomogram from our NRHAD cohort, with a C index of 0.935 (95% CI, 0.910–0.967). The linear predictor corresponding to the cumulative points indicated the different possibilities for AD-free survival (shown in the figure under the years of follow-up).

### Baseline demographic and clinical characteristics of participants from the CABLE cohort.

The demographic and clinical characteristics of the participants from the Chinese Alzheimer’s Biomarker and Lifestyle (CABLE) cohort baseline data are shown in Supplemental Table 3. Similar to participants in the ADNI and NRHAD databases, there were no significant differences in age or sex among the 3 groups. We observed a statistically significant difference in the years of education between the 3 groups (*P* < 0.05). MMSE and MoCA assessments showed that both AD (*P* < 0.001) and MCI (*P* < 0.001) patients had lower cognitive function than did HCs. Moreover, there were more ApoEε4 carriers among the patients with MCI or AD than among the HCs (*P* < 0.001).

### The predictive value of CSF sTREM2 levels in progression to AD in the CABLE cohort.

No statistically significant differences in age, sex, ApoE ε4–carrier genotype, or years of education were observed between the MCI-nc, whose cognitive function was stable during follow-up, and MCI-c, who progressed to AD during follow-up (Supplemental Table 4). We further validated the predictive value of CSF sTREM2 levels in progression to AD in the CABLE cohort in a longitudinal study. After adjusting for age, sex, and p-Tau/t-Tau ratios, we used the Cox proportional hazards model to evaluate the predictive value of CSF sTREM2 levels in the conversion process. The results showed that patients with MCI who had CSF sTREM2 levels below 18,000 pg/mL (HR = 3.65, *P* = 0.034, Supplemental Table 4) and CSF Aβ_1–42_ levels below 215.0 pg/mL (HR = 3.84, *P* = 0.027, Supplemental Table 4) were more likely to develop AD.

There were no statistical differences between HCs who were amyloid deposition–positive (A^+^) and HCs who were amyloid deposition–negative (A^–^) (Supplemental Figure 2A). Among the MCI groups, CSF sTREM2 levels were significantly higher in patients who were A^+^ than in those who were A^–^ (Supplemental Figure 2A, *P* < 0.01), whereas CSF sTREM2 levels were further increased in patients with AD who were A^+^ (Supplemental Figure 2A, *P* < 0.05) after adjusting for age, sex, and the p-Tau/t-Tau ratio. Kaplan-Meier curves were used to evaluate the predictive value of CSF sTREM2 in the conversion process. As shown in Supplemental Figure 2B, patients with MCI whose CSF sTREM2 levels were below 18,000 pg/mL had a mean AD-free survival of 24 months, whereas those with sTREM2 levels of 18,000 pg/mL or higher had a mean AD-free survival of 36 months (*P* = 0.0178). Furthermore, patients with MCI who had CSF sTREM2 levels below 18,000 pg/mL were more likely to develop AD.

### CSF sTREM2 levels are correlated with plasma sTREM2 levels in the CABLE cohort.

**Figure 5:**
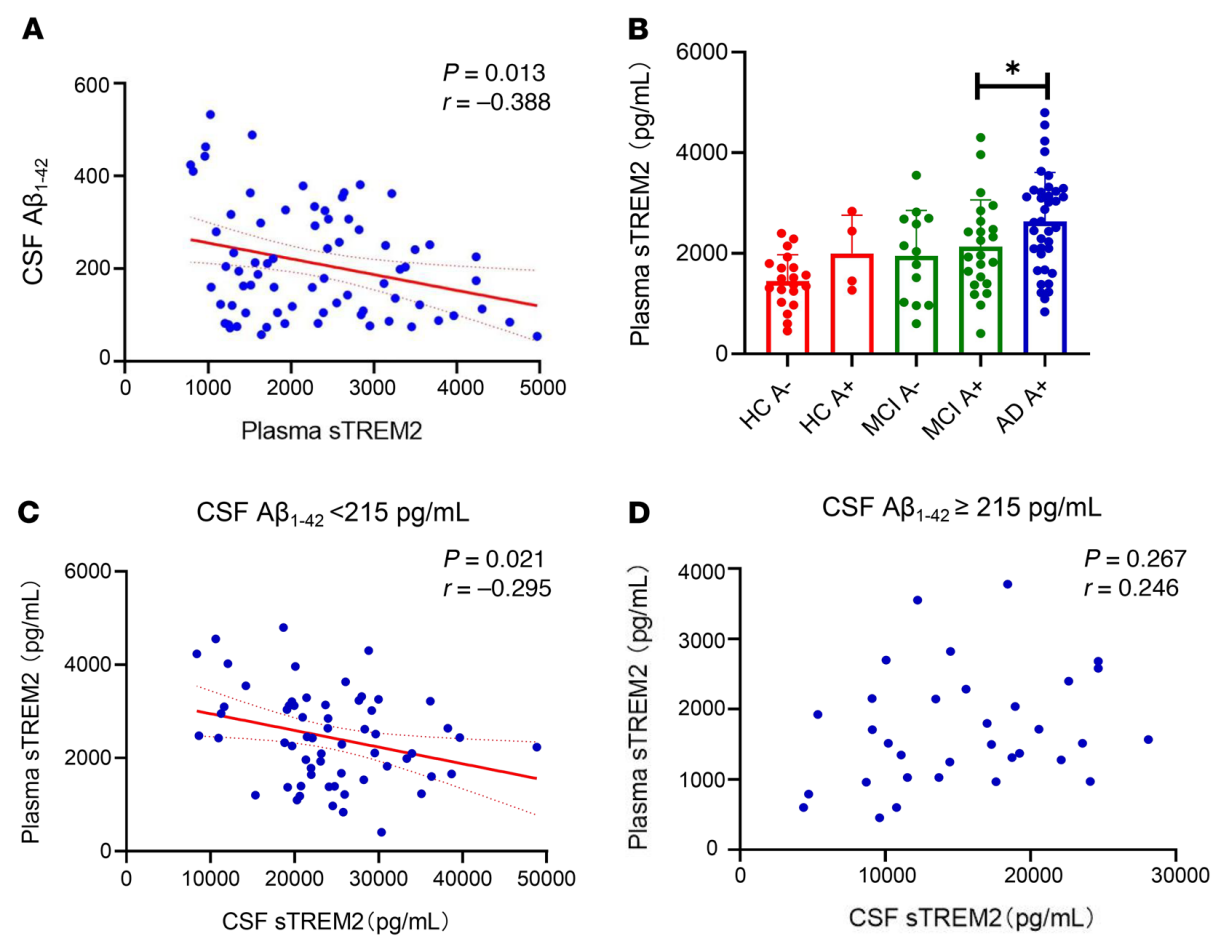
Association between CSF and plasma sTREM2 levels in the CABLE cohort. (**A**) CSF Aβ_1–42_ levels were negatively correlated with plasma sTREM2 levels. (**B**) Plasma sTREM2 levels were significantly increased in the AD group. (**C**) CSF sTREM2 levels were negatively correlated with plasma sTREM2 levels for patients with CSF Aβ_1–42_ levels below 215 pg/mL. (**D**) CSF sTREM2 levels were uncorrelated with plasma sTREM2 levels in patients with CSF Aβ_1–42_ levels of 215 pg/mL or higher. Data represent the mean ± SEM. A total of 50 HCs, 50 patients with MCI, and 50 patients with AD were included for analysis in CABLE cohort. *P < 0.05, by 1-way ANOVA with the least significant difference post hoc test and Pearson’s correlation test.

Next, we investigated the correlation between CSF sTREM2 and plasma sTREM2 levels as well as between CSF Aβ_1–42_ and plasma sTREM2 levels in the CABLE cohort. The results showed that CSF Aβ_1–42_ levels were negatively correlated with plasma sTREM2 levels (Figure 5A). The levels of plasma sTREM2 in the MCI groups were higher than those in the HC groups and were further increased in the AD groups (Figure 5B). To determine the association between CSF sTREM2 and plasma sTREM2 levels, we analyzed the correlation on the basis of CSF Aβ_1–42_ levels. We found that CSF sTREM2 levels were negatively correlated with plasma sTREM2 levels when CSF Aβ_1–42_ was less than 215 pg/mL, while they were uncorrelated with plasma sTREM2 when CSF Aβ_1–42_ levels were greater than 215 pg/mL (Figure 5, C and D, and Supplemental Figure 3).

## Discussion

In this study, we found that (a) in the ADNI and CABLE database patients with MCI who had positive amyloid deposition and low CSF Aβ_1–42_ concentrations, CSF sTREM2 levels were inversely associated with the risk of AD; (b) in the NRHAD clinical cohort, plasma sTREM2 levels in the AD group were higher than those in the HC and MCI groups; (c) in the NRHAD database patients with MCI who had low plasma Aβ_1–42_ concentrations, higher plasma sTREM2 levels were associated with a shorter conversion time from MCI to AD in our NRHAD database; and (d) CSF sTREM2 levels were correlated with plasma sTREM2 levels in the CABLE database cohort. Moreover, our findings suggest that both CSF and peripheral plasma sTREM2 levels are associated with amyloid levels in the MCI and AD patient groups.

In the present study, we found no significance difference in CSF sTREM2 levels between the groups in the ADNI database, whereas CSF sTREM2 levels were significantly increased in patients with AD in the CABLE database. However, the nature of AD-related sTREM2 alterations remains debatable. A previous small-sample study reported lower levels of sTREM2 in patients with AD or frontotemporal dementia than in HCs (5). In contrast, a separate study of a larger population reported significantly higher levels of CSF sTREM2 in individuals with AD or frontotemporal dementia than in cognitively normal controls (16). A recent meta-analysis showed that CSF sTREM2 levels increased in early AD stages and then decreased slightly at the dementia stage (27). Therefore, the discrepancy between the present and previous findings may be due to differences in participants’ characteristics, including their stage of AD.

A recent study showed an association between high CSF sTREM2 concentrations and a reduced rate of memory decline in individuals with MCI or AD dementia who were positive for CSF Aβ_1–42_ and CSF p-Tau (Thr181) (5). In patients with autosomal dominant AD, elevated levels of CSF sTREM2 were observed in the brain after amyloid deposition and neuronal injury and 5 years before the expected onset of symptoms (16). In addition, in patients with MCI, CSF sTREM2 levels were positively associated with gray matter volume in the bilateral inferior and middle temporal cortices, the precuneus, and the supramarginal and angular gyri, whereas these levels were negatively correlated with the mean diffusivity values (28). These findings provide evidence for the predictive value of CSF sTREM2 levels in MCI-to-AD conversion. Therefore, to further validate these findings, we performed a longitudinal study of the ADNI and CABLE databases and found that high levels of CSF sTREM2 were associated with a decreased risk of MCI-to-AD conversion. Our longitudinal study revealed a statistically significant correlation after adjusting for p-Tau/t-Tau values (5, 28).

Early diagnosis and identification of preclinical AD is paramount for optimal patient outcomes. Hence, we explored the role of the soluble form of TREM2 in plasma. The levels of sTREM2 in peripheral blood were elevated in our clinical MCI and AD patient groups; these findings are consistent with those of a previous study involving patients with AD (23). In addition, plasma Aβ_1–42_ concentrations were positively correlated with plasma sTREM2 levels at both the MCI and AD stages. Several cross-sectional studies demonstrated that higher TREM2 levels in PBMCs were associated with MCI-to-AD conversion (22–24). Our longitudinal study further confirmed the predictive value of plasma sTREM2 levels, showing that elevated plasma sTREM2 levels could be a potential predictive biomarker of MCI-to-AD conversion.

CSF sTREM2 levels reflect the microglial activation state in response to neurodegeneration, in which lower CSF sTREM2 levels could result in faster progression of the disease due to decreased microglial clearance of intracerebral Aβ (11–13, 29). Although the relationship between plasma sTREM2 levels and the peripheral immune response in AD is poorly understood, plasma sTREM2 primarily originates from macrophages (30), therefore, higher plasma sTREM2 levels might reflect the activation of peripheral macrophages, which could aggravate the MCI-to-AD conversion. Given the insufficient CSF sample size in the NRHAD database, we did not analyze the predictive value of CSF sTREM2 levels or the correlation between CSF and plasma levels of sTREM2, and proceeded instead to investigate the predictive value of CSF sTREM2 levels in the CABLE cohort. In the CABLE cohort, we found that CSF sTREM2 levels were highly correlated with plasma sTREM2 levels. These findings provide additional evidence that peripheral sTREM2 levels are correlated with central sTREM2 levels and that plasma sTREM2 levels could be a useful biomarker for MCI-to-AD conversion.

The present study has some limitations that should be considered when interpreting the findings. First, the accuracy of Aβ_1–42_ measurements was compromised by the limits of sensitive detection. Second, it remains to be determined whether the changes in CSF sTREM2 levels are consistent with the altered expression of cerebral TREM2 and TREM2-related microglial activation. However, the functional relationship between CSF sTREM2 levels and microglial TREM2 activation remains unclear. Third, sTREM2 signaling interacts with other inflammatory pathways, leading to an altered peripheral inflammatory response. Fourth, although most AV45 SUVR findings in patients with AD were consistent with their cognitive symptoms, some patients with dementia were negative for amyloid deposition. Finally, the different detection kits used in the study showed variable levels of plasma sTREM2 and CSF sTREM2 in the different cohorts. It would be worthwhile in the future to recruit more individuals to verify the accuracy of sTREM2 levels in predicting MCI-to-AD conversion using the same commonly available commercial kit brand.

In conclusion, we found that elevated CSF sTREM2 levels and reduced plasma sTREM2 levels were associated with delayed progression from MCI to AD, suggesting that sTREM2 levels may be a biomarker for the risk of MCI-to-AD conversion.

## Methods

### Study design.

A total of 763 HCs, 971 patients with MCI, and 375 patients with AD were included in the ADNI database (Caucasian cohort) from 2010 to 2018 were included in the present analysis. In the ADNI database, AD was diagnosed according to the National Institute of Neurological Disorders and Stroke–Alzheimer Disease and Related Disorders (NINCDS-ADRDA) criteria for probable AD (25), whereas MCI was diagnosed according to the Mayo Clinic criteria (31). Soluble Aβ_1–42_ peptide forms present in the CSF were quantified, and the fibrillary forms were evaluated by AV45 imaging (26). In addition to the ADNI criteria, we applied the following exclusion criteria: (a) absence of TREM2 variants (HCs = 3, MCI = 10, AD = 9); (b) availability of CSF sTREM2 samples (HCs = 528, MCI = 463, AD = 182); CSF Aβ_1–42_ level estimates (HCs = 4, MCI = 3, AD = 2) and AV45 imaging data at baseline (HCs = 82, MCI = 125, AD = 71); and (c) follow-up evaluation of cognitive ability of patients with MCI after a minimum of 12 months. After excluding the individuals with MCI who were lost to follow-up (MCI = 24) and who died (MCI = 5), a total of 146 HCs, 344 patients with MCI, and 111 patients with AD were included for further analysis (Figure 1). A cognitive assessment was carried out every 6 months, and deaths were excluded from the AD progression-free survival outcomes data. In this study, we cross-sectionally analyzed the levels of sTREM2 and Aβ_1–42_ in the CSF, the AV45 imaging findings, and the SUVRs as possible predictors of longitudinal cognitive progression in patients with MCI.

For our NRHAD database (China cohorts), participants were recruited from the neurology clinic at Ruijin Hospital between October 2015 and May 2019. All patients with AD were diagnosed with probable AD dementia on the basis of the National Institute on Aging and Alzheimer’s Association’s diagnostic guidelines, using structural MRI scans (32). MCI with deficits in memory function was diagnosed according to the Mayo Clinic criteria (33, 34). These criteria include subjective memory complaints corroborated by an informant, together with preserved everyday activities, memory impairment based on a standard neuropsychological test, preserved global cognitive functions, and the exclusion of dementia. The HCs were matched for age, sex, and education attainment and recruited from the local community in Shanghai. Inclusion criteria for HCs included an MMSE score of 28 or higher with no memory-related complaints. In addition, participants were excluded if they met any of the following criteria: diagnosis of another neurological disease or diabetes mellitus, history of acute cerebrovascular or cardiovascular accident, ongoing systemic disorders such as malignancy or lupus, severe infection, drug abuse or dependency, and severe psychiatric disorders (35).

Experienced neurologists performed all diagnostic assessments based on a thorough review of the patients’ medical history, neuropsychological testing battery, neurological examinations, laboratory tests, and structural MRI findings. The ApoE genotype was determined as previously described (36). Follow-up visits started in October 2016 and continued until May 2019, with a mean follow-up period of 34.7 months. Neuropsychological tests were performed every 12 months to evaluate the participants’ cognitive ability. During the follow-up period, patients with MCI were categorized as MCI-c or MCI-nc.

For the CABLE database (China cohorts), 50 patients with MCI, 50 patients with AD, and 50 HCs, were recruited from Qingdao Municipal Hospital for the present analysis (37). The inclusion and diagnostic criteria for the HCs and individuals with MCI or AD were the same as those for the NRHAD database. Experienced neurologists performed all the diagnostic assessments and neuropsychological tests. The mean follow-up period was 48 months. In addition, neuropsychological tests were performed at every 12-month follow-up visit to evaluate the participants’ cognitive abilities. Patients were categorized as MCI-c or MCI-nc, depending on whether they progressed to AD during the follow-up period.

### Measurements of sTREM2 and Aβ_1–42_ levels (ADNI database).

A detailed description of CSF sTREM2 and Aβ_1–42_ data as well as the original results can be downloaded from the USC Laboratory of Neuro Imaging (LONI) Image and Data Archive (IDA) (https://ida.loni.usc.edu). Measurements of CSF Aβ_1–42_ levels were performed by the ADNI Biomarker Core team at the University of Pennsylvania (Philadelphia, Pennsylvania, USA) using Elecsys immunoassays and fully automated Elecsys Cobase 601 instruments (provided as a UPENNBIOMK9.csv file by the ADNI databank; Elecsys). The measurement range (lower to upper technical limit) was 200 to 1,700 pg/mL for Aβ_1–42_. The CSF sTREM2 measurements, adjusting for p-Tau/t-Tau values, were based on the Meso Scale Discovery (MSD) platform established by Haass and colleagues as previously reported (19), and the CSF sTREM2 values were corrected on the basis of values of the 4 internal standards (ISs), which were loaded on all plates (the results can be found under the name “MSD_sTREM2CORRECTED.csv” in the ADNI database).

### Evaluation of fibrillar amyloid by AV45 (ADNI database).

Florbetapir synthesis and image acquisition details are described in the ADNI database (https://ida.loni.usc.edu) (26). Florbetapir images consisted of four 5-minute frames acquired 50–70 minutes after injection and were realigned, averaged, resliced to a common voxel size (1.5 mm^3^), and smoothed to a common resolution of 8 mm^3^ in full width at half maximum (38). To define cortical regions and the reference regions of interest, structural T1 data were simultaneously acquired as a structural template using FreeSurfer software (version 4.5.0; surfer.nmr.mgh.harvard.edu) (39). Baseline florbetapir scans were used to extract SUVRs within 4 large cortical regions of interest (frontal, cingulate, parietal, and temporal cortices), which were averaged to create the SUVR (26).

### Quantification of sTREM2 and Aβ_1–42_ levels (clinical cohort).

All blood samples were centrifuged, and the supernatant was stored in separate vials at –80°C for batch plasma analysis. Commercially available ELISAs were performed in duplicate to estimate plasma sTREM2 levels for the NRHAD data, according to the manufacturer’s instructions (RayBiotech). T-Tau, p-Tau (Thr181), and Aβ_40_ and Aβ_42_ levels were measured using the associated kits (MilliporeSigma) and following the manufacturer’s instructions for Luminex XMap technology on the Luminex 100 platform. Commercially available ELISA assays were used to estimate CSF and plasma sTREM2 levels in the CABLE analysis (37) according to the manufacturer’s instructions (Abcam). CSF p-Tau, t-Tau, and Aβ_40_ and Aβ_42_ levels were measured using the associated kits (Innotest, Fujirebio) with a microplate reader (Multiskan MK3; Thermo Fisher Scientific).

### Statistics.

Statistical analysis was performed using SPSS (version 19.1; IBM Corp.) and R (version 3.4.0; R Development Core Team 2017). The significance level for all tests was set at a *P* value of less than 0.05. The cutoff value for the AV45 SUVR was 1.11, using the whole cerebellum reference region (40). One-way ANOVA with the least significant difference post hoc test was used to compare differences among the HC, MCI, and AD groups and their corresponding subgroups. We used χ^2^ and split χ^2^ tests to identify among-group differences in dichotomous variables, such as sex and ApoE ε4 carrier status. Pearson’s correlation test was used to determine the association between the AV45 SUVR and sTREM2 and Aβ_1–42_ levels in both CSF and plasma after adjusting for other covariants. The rate of progression-free survival was estimated using the Kaplan-Meier method in the present study, and any differences in survival were evaluated using a stratified log-rank test. Multivariable analyses with the Cox proportional hazards model were used to evaluate the risk of MCI conversion during the follow-up period (based on AV45 SUVR, sTREM2, and Aβ_1–42_ values estimated from both CSF and plasma). All data analyzed using the Cox proportional hazards model were adjusted for the p-Tau/t-tau ratio, age, and sex. The LASSO algorithm was used to select relevant biomarker features and build a predictive model for the AD biomarker profiles. All biomarker variables were included in the model, together with the variables used in the reference model (age, sex, years of education, and ApoE ε4 carrier status) that were tested independently. The CSF and plasma biomarkers were tested separately. A 10-fold cross-validation process was performed for each LASSO analysis using the glmnet package (41), which allowed estimation of the CI of the misclassification error for each value of the regularization parameter. The LASSO analysis was repeated 100 times. A model that minimized the cross-validated misclassification error across 100 runs was selected. Its performance was assessed by receiver operating characteristic (ROC) AUC estimation (using a bootstrap approach with 1,000 iterations) and compared with the reference model’s AUC and accuracy (using the McNemar test). The cutoff values for CSF Aβ_1–42_ and CSF sTREM2 levels were determined using the area under the ROC curve (AUC), sensitivity, and specificity values in the ROC analysis. The optimal cutoff point value was the point minimizing the summation of the absolute values of the differences between AUC and sensitivity or specificity, which provided that the difference between sensitivity and specificity was minimal.

### Study approval.

For our NRHAD database, participants were recruited from the neurology clinic at Ruijin Hospital from October 2015 to May 2019. All volunteers provided informed, written consent before study participation. This study was approved by the Research Ethics Committee of Ruijin Hospital. For the CABLE database, all volunteers provided informed, written consent before study participation. The IRB of Qingdao Municipal Hospital approved the use of the CABLE cohort data for this study (37).

## Author contributions

JL designed the study, provided financial support, and revised the manuscript. YJ and YD revised the manuscript. YL provided financial support. AZ, GY, and WK collected the data. AZ performed data analysis. AZ and YJ wrote the manuscript. LT provided the CABLE database samples. All the co-authors contributed to revising the manuscript for intellectual content and approved the final version for publication.

## Supplementary Material

Supplemental data

ICMJE disclosure forms

## Figures and Tables

**Table 1:**
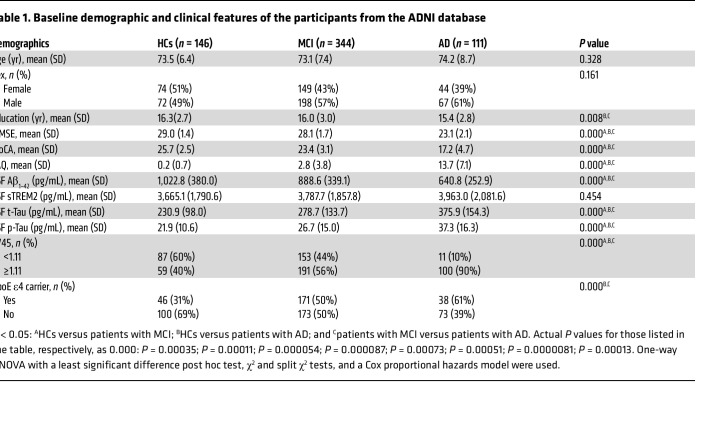
Baseline demographic and clinical features of the participants from the ADNI database

**Table 2:**
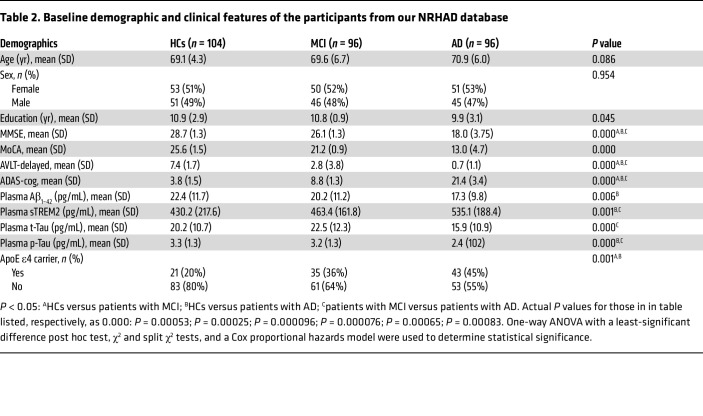
Baseline demographic and clinical features of the participants from our NRHAD database

## Version 1 12/15/2022

Electronic publication
